# Evaluation of the Efficiency of the Reticulocyte Hemoglobin Content on Diagnosis for Iron Deficiency Anemia in Chinese Adults

**DOI:** 10.3390/nu9050450

**Published:** 2017-05-02

**Authors:** Jie Cai, Meng Wu, Jie Ren, Yali Du, Zhangbiao Long, Guoxun Li, Bing Han, Lichen Yang

**Affiliations:** 1 The Key Laboratory of Trace Element Nutrition, National Institute for Nutrition and Health, Chinese Center for Disease Control and Prevention, 29 Nan Wei Road, Xicheng District, Beijing 100050, China; caijie1113@126.com (J.C.); wumeng0208@126.com (M.W.); 2 Health and Family Planning Supervision Institution of Chaoyang, Panjiayuan Huawei 25, Chaoyang District, Beijing 100021, China; jieren0111@163.com; 3 Department of Hematology, Peking Union Medical College Hospital, Chinese Academy of Medical Sciences and Peking Union Medical College, Beijing 100730, China; yali_crazy@126.com (Y.D.); longzhangbiao@163.com (Z.L.); hanbing_li@sina.com.cn (B.H.); 4 Department of General Surgery, Tianjin People’s Hospital, 190, Jieyuan Road, Hongqiao District, Tianjin 300121, China; liguoxun@126.com

**Keywords:** reticulocyte hemoglobin content, iron deficiency anemia

## Abstract

Our aim was to evaluate the cut-off value and efficiency of using reticulocyte hemoglobin content as a marker to diagnose iron deficiency anemia in Chinese adults. 140 adults who needed bone marrow aspiration for diagnosis at the hematology department of the Peking Union Medical College Hospital were enrolled according to the inclusive and exclusive criteria. Venous blood samples were collected to detect complete blood count, including hemoglobin, reticulocyte hemoglobin content, hematocrit, mean cellular volume, corpuscular hemoglobin concentration, hemoglobin content, free erythrocyte protoporphyrin; iron indexes of serum ferritin, serum transferrin receptor, and unsaturated iron-binding capacity; and inflammation markers of C-reactive protein and α-acid glycoprotein. Bone marrow samples were obtained for the bone marrow iron staining, which was used as the standard for the evaluation of iron status in this study. Subjects were divided into three groups according to hemoglobin levels and bone marrow iron staining results: the IDA (iron deficiency anemia) group, the NIDA (non-iron deficiency anemia) group, and the control group. The differences of the above-mentioned indexes were compared among the three groups and the effect of inflammation was also considered. The cut-off value of reticulocyte hemoglobin content was determined by receiver operation curves. The IDA group (*n* = 56) had significantly lower reticulocyte hemoglobin content, mean cellular volume, corpuscular hemoglobin concentration, hemoglobin content, and serum ferritin; and higher free erythrocyte protoporphyrin, unsaturated iron-binding capacity, and serum transferrin receptor (*p* < 0.05) compared with the NIDA group (*n* = 38) and control group (*n* = 46). Hematocrit, serum ferritin, and unsaturated iron-binding capacity were significantly affected by inflammation while reticulocyte hemoglobin content and other parameters were not. The cut-off value of reticulocyte hemoglobin content for diagnosing iron deficiency anemia was 27.2 pg, with a sensitivity of 87.5% and a specificity of 92.9%. The cut-off values for mean cellular volume, serum ferritin, and serum transferrin receptor were 76.6, 12.9, and 4.89 mg/L, respectively. Reticulocyte hemoglobin content had the largest area under the curve of 0.929, while those for mean cellular volume, serum ferritin, serum transferrin receptor were 0.922, 0.887, and 0.900, respectively. Reticulocyte hemoglobin content has a high sensitivity and specificity in the diagnosis of iron deficiency anemia, and its comprehensive diagnostic efficacy is better than other traditional indicators—such as serum ferritin and serum transferrin receptor.

## 1. Introduction

Anemia remains a highly prevalent global health problem, which affects 43% of children younger than five years old, 38% of pregnant women, and 29% of non-pregnant women worldwide [1]. Iron deficiency is the most common etiology globally [2]. Iron deficiency anemia (IDA) can lead to developmental delays, behavioral disturbances, perinatal complications, and the impairment of learning ability and cognitive function [3,4], and early detection of IDA is essential in helping to prevent various complications and for improving patient quality of life [5]. A variety of tests are used for evaluating anemia, and at present the most reliable method is a bone marrow biopsy with Prussian blue staining. However, the drawbacks of bone marrow examination—cumbersome, expensive, and traumatic—make it unsuitable for routine use. In addition, the sensitivity and specificity to many indicators, such as serum ferritin (SF) and serum transferrin receptor (sTfR), are far from satisfactory [5,6].

Fortunately, several studies have indicated that reticulocyte hemoglobin content (CHr) seems to be a good predictor of IDA [7,8,9]. Reticulocytes are made in the bone marrow and released into the bloodstream, where they circulate for about one or two days before developing into mature red blood cells. Compared with erythrocytes, the shorter lifespan of reticulocytes makes CHr a better biomarker to reflect iron status in the short term [10]. It gives a snapshot of the iron availability for erythropoiesis [11]. Additionally, CHr has a high specificity for not being affected by inflammation, and also exhibits a low coefficient of variation [12]. Compared with the traumatic bone marrow biopsy, it is also relatively cheap, convenient, and less invasive because only several milliliters of peripheral blood are needed to get CHr data. All of these characteristics make CHr a suitable alternative when the ‘gold standard’ of bone marrow examination is limited. Although CHr is currently considered as a preferable biomarker of IDA, the systematic differences of measurement and the lack of consistent cut-off value make it necessary to further explore its significance for clinical application [13], and studies focusing on Chinese adults are still insufficient. Additionally, bone marrow iron staining results were seldom used as the group criterion in similar studies because of the difficulty in collecting subjects with a result for it. Most of them used serum ferritin combined with other markers instead. In this study, we aimed to take the bone marrow iron staining result as the gold standard, to compare the efficiency of CHr, SF, sTfR and other indexes in the diagnosis of IDA, and explore the suitable cut-off values.

## 2. Materials and Method

### 2.1. Location and Experimental Design

The study was carried out in the Peking Union Medical College Hospital and National Institute of Nutrition for Health, Chinese Center for Disease Control and Prevention. The collection of subjects was between January 2015 and June 2016. 140 patients who needed to undergo a bone marrow aspiration for diagnosis in the hematology department of Peking Union Medical College Hospital were enrolled following the informed consent and ethical review. Basic information was first collected, such as name, age, gender, contact method, primary diagnosis and consultation time. Inclusion criteria were as follows: (1) subjects had to be adults (not younger than 18 years old); (2) subjects needed to undergo a bone marrow aspiration for diagnosis. Exclusion criteria were: (1) pregnant women; (2) subjects who had a blood transfusion, oral, or intravenous iron supplement within a month; (3) patients who suffered from hemoglobinopathy, leukemia, myeloma, or myelodysplastic syndrome. Venous blood samples were collected to detect complete blood count, including hemoglobin (Hb), CHr, hematocrit (HCT), mean cellular volume (MCV), mean corpuscular hemoglobin concentration (MCHC), hemoglobin content (CH), free erythrocyte protoporphyrin (FEP), iron indexes of serum ferritin (SF), serum transferrin receptor (sTfR) and unsaturated iron-binding capacity (UIBC), and inflammation markers of C-reactive protein (CRP) and α-acid glycoprotein (α-AGP). Bone marrow samples were obtained for the bone marrow iron staining. 140 eligible subjects were divided into three groups according to Hb levels and bone marrow iron staining results: (1) The IDA group: bone marrow iron staining showed negative, Hb < 110 g/L (female) and Hb < 120 g/L (male); (2) The NIDA (non-iron deficiency anemia) group: bone marrow iron staining showed positive, Hb < 110 g/L (female) and Hb < 120 g/L (male); (3) The control group: bone marrow iron staining showed positive, Hb ≥ 110 g/L (female) and Hb ≥ 120 g/L (male).

### 2.2. Analytical Method

Venous blood samples were collected by evacuated tubes containing K2-EDTA (BD, Franklin Lakes, NJ, USA). A centrifugal speed freezing centrifuge (KDC-2046, Zhongke Zhongjia, Hefei, China) was used to centrifuge blood samples. A complete blood cell count (Hb, CHr, HCT, MCV, MCH, MCHC, CH, and FEP) was measured by an automatic blood cell analyzer (ADVIA120, Bayer, Leverkusen, Germany). Bone marrow smears were obtained from the bone marrow aspiration of the subjects. Prussian blue staining was used to observe the iron in the reticulocytes. The results for “+/++/+++” were defined as positive results and “-” was defined as negative. These analyses were performed at the laboratory department of the Peking Union Medical College Hospital. Biochemical assays of iron status (sTfR, SF, UIBC) and inflammation markers (CRP, α-AGP) were performed using an automatic biochemical analyzer (7180 Hitach, Ibarakiken, Japan) at the Key laboratory of Trace Element Nutrition of the Ministry of Health, National Institute of Nutrition for Health, Chinese Center for Disease Control and Prevention. All test reagents were purchased from Roche.

### 2.3. Statistical Analysis

All data were entered into a Microsoft Excel sheet and double-checked. General statistical analysis was performed using SPSS version 19.0. Receiver Operation Curves (ROCs) and cut-off value analysis were performed using MedCalc 13.0. The normality of the data distribution of the tests under study was investigated with the Kolmogorov-Smirnoff test. FEP, SF, and sTfR did not obey normal distribution, so they were expressed as median (P25, P75). Other quantitative variables were expressed as means ± standard deviations ($\bar{X}\pm SD$). The comparison of the differences among the groups was analyzed using one-way analysis of variance (ANOVA). The differences between the two groups were compared by *t* test. Although FEP, SF, and sTfR did not follow the normal distribution, their logarithms did. So logarithmic transformation was conducted on them before the ANOVA and *t* test. *p* values less than 0.05 were considered statistically significant. ROC analysis was used to evaluate the diagnostic efficiency of each index and determine the diagnostic cut-off value of each index with its sensitivity and specificity. In this study, the patients in the IDA group were defined as the positive group.

## 3. Results

The basic information was described in Table 1. Of the 140 subjects studied, 56 (40%) were classified into the IDA group, 38 (27%) into the NIDA group, and 46 (33%) into the control group. There was no significant difference in age among the three groups (*p* > 0.05), and the mean age of all subjects was 45 years old, ranging between 20 and 84. While the gender composition showed significant difference among the three groups (*p* < 0.05), the proportion of female in the IDA group was higher than other two groups. The total ratio of male:female was 0.4:1. The main diagnosis of NIDA group included lymphoma, megaloblastic anemia, hemolytic anemia, diopathic aplastic anemia, and anemia of chronic disease. The main diagnosis of the control group included thrombocytopenia and leukopenia. 

Mean values or medians of different biochemical and hematological parameters in each group are presented in Table 2. Significant differences were found in all parameters among the three groups. The mean value of CHr for the IDA group was 23.3 ± 4 pg, significantly lower (*p* < 0.05) than that in the NIDA and control groups. For other parameters, the IDA group had significantly lower MCV, MCHC, CH, SF; and higher FEP, UIBC, sTfR compared with the other two groups (*p* < 0.05).

We also divided the subjects with anemia (IDA group and NIDA group) into two groups according to the inflammatory indexes. The group without inflammation included people whose CRP ≤ 5 mg/L and AGP ≤ 1 g/L, and individuals would be regarded as inflammation infected as long as one indicator rose. Of the 94 people studied, 24 (26%) were infected with inflammation and 66 (70%) were not, while there is no data about inflammation for the remaining 4%. The biochemical and hematological parameters of the two groups are presented and compared in Table 3. HCT, UIBC, and SF differed significantly (*p* < 0.05) between two groups; people with inflammation had lower HCT, UIBC, and higher SF. In particular, the median of SF in the group with inflammation was nearly 28 times that of the group without inflammation. Other parameters—including CHr, Hb, MCV, MCHC, CH, UIBC, FEP and sTfR—showed no significant difference between the two groups.

**Figure 1:**
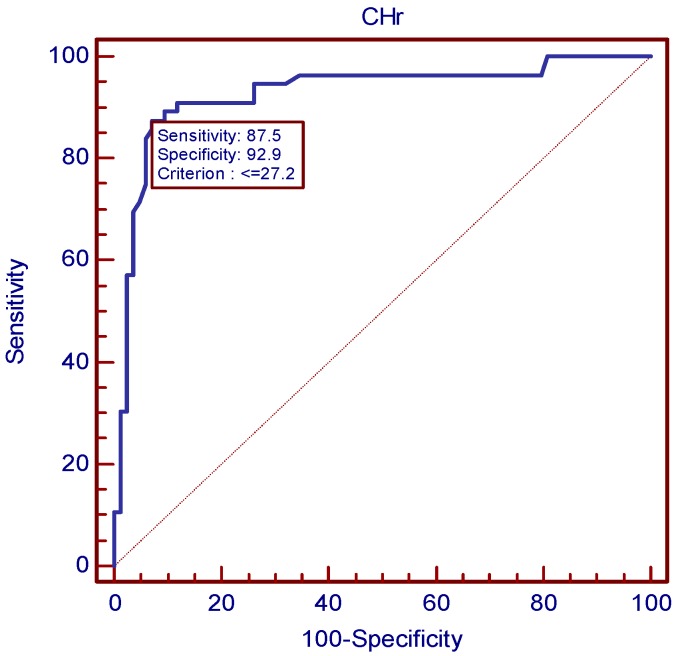
ROC curve of CHr diagnosing for IDA.

**Figure 2:**
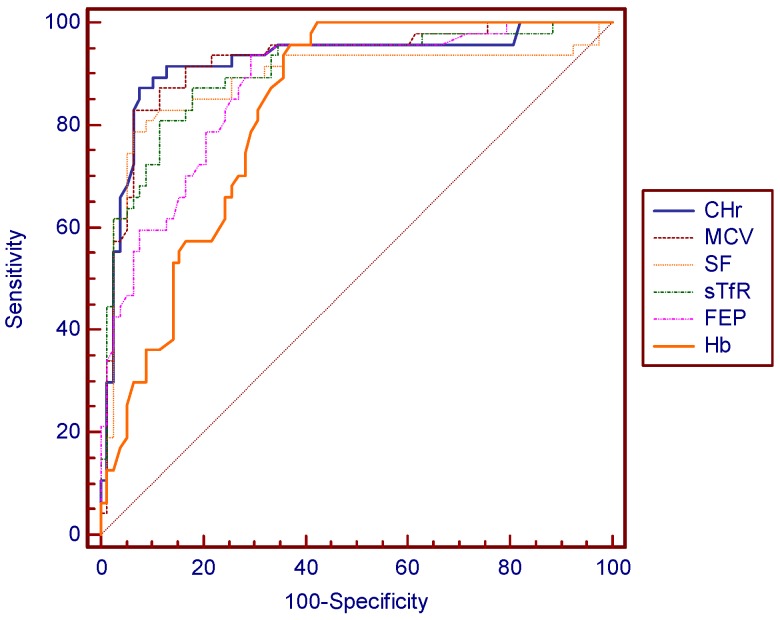
Comparison of ROC curves of CHr, MCV, SF, sTfR, FEP, and Hb diagnosing for IDA.

Cut-off values for best sensitivity and specificity were acquired using ROC (Figure 1, Table 4). According to ROC, the best cut-off point of CHr for diagnosing IDA was 27.2 pg, corresponding to a sensitivity of 87.5% and a specificity of 92.9%, respectively. CHr values of ≤18.3 pg/L and ≥34.2 pg/L had sensitivity and specificity of 100%. The positive predictive value of CHr is 89.1% and negative predictive value is 91.8%. A MCV value of 76.6 mg/L had a sensitivity of 83.9% and a specificity of 92.9%. A SF value of 12.9 mg/L had a sensitivity of 79.2% and a specificity of 93.9%. A sTfR value of 4.89 mg/L had a sensitivity of 86.8% and a specificity of 81.7%. Although there is no significant difference in the areas under curves (AUC) for these indexes except for Hb, CHr still showed a slender superiority (Figure 2). 

## 4. Discussion

The gender composition showed significant difference among the three groups, while age did not. The proportion of female in the IDA group was significantly higher than that of other groups. This result may be explained by the fact that women are at higher risk for IDA because of the monthly loss of iron with menstrual bleeding [14].

Compared with the NIDA group and the control group, the IDA group had significantly lower CHr, MCV, MCHC, CH, and SF; and higher FEP, UIBC and sTfR. When the loss of iron exceed the intake, a negative iron balance appears resulting in iron deficiency anemia. Continued iron loss finally impairs hemoglobin synthesis and causes mild normocytic anemia, with a normal MCV [14]. Low SI and sTfR and increased UIBC may appear at this stage. With the progress of the condition, a more severe microcytic anemia eventually comes, with low CHr, MCV, MCHC, CH, and SF; and high FEP, UIBC, and sTfR. As a young stage red blood cell, with a short lifespan of only 24 to 48 h [10,15], reticulocytes are more sensitive to iron deficiency and can be used to detect it earlier on compared with parameters such as SF and Hb.

Many indexes of iron status are affected by inflammation. The WHO and the United States CDC experts also stress that inflammatory indicators need to be detected when detecting indexes of iron nutritional status [16]. Studies have shown that AGP and CRP are significantly correlated with SF, and it is suggested to detect AGP and CRP simultaneously when assessing the nutritional status of iron [17]. Therefore, we grouped the subjects according to AGP and CRP to examine the effect of inflammation on the diagnostic accuracy of CHr. There was no significant difference in CHr between patients with or without inflammation, which means inflammation would not affect the diagnostic efficiency of CHr. In contrast, SF is significantly elevated (nearly 28 times) in patients with inflammation. This result, in accordance with other studies [7,17], makes CHr a more accurate and convenient indicator to diagnosis IDA independently, without the assistance of inflammation parameters. 

The diagnostic efficiency of IDA diagnosed by CHr, MCV, sTfR, and SF were compared by using the AUC of ROCs. CHr was found to have the best diagnostic efficiency (AUC of 0.929), indicating its superior ability of diagnosing IDA over other indexes. The diagnostic sensitivity and specificity of CHr both reached a relatively ideal level. They were both higher than that of sTfR. Even when sensitivity was higher in other markers, the sensitivity was very low and vice versa. When CHr was taken as a diagnosing marker, the sensitivity and specificity reached a good balance. The positive predictive value of CHr is the highest, and the negative predictive value also reached a relatively ideal level. In general, CHr performed best in the accuracy of the diagnosis for IDA. Nowadays, besides the gold standard, the most commonly used clinical indicator for reflecting iron status is SF [14], and a cut-off value of 12 g/L has been widely used for adults, denoting a depletion of iron stores [18]. However, serum ferritin level often increases independently, affected by factors such as acute or chronic inflammation, infection, liver disease, malignancy, and alcohol use [19]. Unlike a potentially traumatic examination, obtaining a CHr result requires only several milliliters of peripheral blood. In addition, CHr is not affected by inflammation, which makes it a better indicator than SF, suggesting that CHr has the potential to be a substitute for SF in diagnosing IDA. However, its specificity is limited by some other conditions, such as thalassemia and anemia of chronic disease. The ratio of microcytic to hypochromic RBC count may help us to distinguish it from thalassemia [20], and the sTfR index will help to distinguish between IDA and anemia of chronic disease [4]. The study showed that CHr has a strong correlation with hemoglobin, MCV, and MCH, meaning that CHr combined with these conventional parameters appears to be available and reliable in identifying IDA [19].

Our study shows that the best threshold for CHr in diagnosing IDA is 27.2 pg. This value is slightly lower than the cut-off points of 28 pg selected by Mast et al. [21], in their 2002 study conducted in America. Natural variation in the human race and changes in life quality and nutrition over several decades may account for this difference. A study by Karlsson et al. [11] obtained a cut-off value of 30.5 pg for CHr, also higher than ours. However, compared with our study, their subjects were all elderly. Mari Rehu et al. [8] reported in 2011 that CHr indexes performed well in differentiating IDA and ACD with a cut-off value of 31.8 pg, and in differentiating IDA and controls with a cut-off value of 30.8 pg. Both of them are higher than our cut-off value of CHr, which may be explained by the difference of subjects and analytical method. The sensitivities were poor when the specificity was above 90% in differentiating IDA and ACD. These three studies also used bone marrow iron as the gold standard. No study of Chinese adults was found in the published research using bone marrow iron staining as a standard. Some other studies often took the serum ferritin as the diagnostic criteria and to obtain the CHr threshold. Eloísa Urrechaga Igartua et al. [22] in 2016 investigated 207 adults and reported that a CHr value of less than 30 pg is a more accurate hematological indicator of iron deficiency compared with all mature erythrocyte related parameters. ID in this study was defined as Hb > 120 g/L (women), >130 g/L (men) and serum ferritin < 30 µg/L. This investigation was made using another analyzer—CellDyn Sapphire (Abbott Diagnostics, Santa Clara, Ca, USA). The systematic differences of measurement and that the target is ID—not IDA—may be the main cause of the difference. That the SF standard used was much higher than the current WHO recommended SF threshold of 12 or 15 g/L might be another cause. Ceylan et al. [23] investigated 109 adults and reported a CHr value of 25.7 pg as the optimal threshold. Their result is lower than that of ours may be explained by the different criteria for IDA and subjects. 

Our study also produced cut-off values of other parameters, especially of SF. The SF value of 12.9 mg/L demonstrated the best threshold for screening IDA. The clinical diagnostic cut-off concentration of SF that is diagnostic varied between 12 and 15 mg/L [24]. Our results are consistent with the current international diagnostic criteria, which indirectly increases the persuasiveness of the CHr threshold obtained.

When evaluating the iron status of an individual, it is favorable to use the fewest, cheapest, and least invasive tests which could accurately reflect iron status. CHr is an inexpensive and readily available assay [25], and is less likely to cause trauma and or discomfort for a patient. With particular regard for the elderly, if CHr in conjunction with other parameters can be used as a substitute for bone marrow iron stain in the diagnosis of IDA, it will greatly reduce their pain and any associated health risks when conducting the procedure. Even with the same diagnostic efficacy of SF, CHr has advantages in its low cost and convenience, let alone a better diagnosing efficiency. In addition, the results from this study are more convincing due to the gold standard of bone marrow iron staining. However, the fact that CHr is not detected by all automatic blood cell analyzers may be the limit for its application at present. A validation study based on the cut-off value of CHr is currently being undertaken by our group. Its application to different gender and age groups may provide further information in the future. 

## 5. Conclusions

CHr has a high sensitivity and specificity in the diagnosis of IDA, and its comprehensive diagnostic efficacy is better than other traditional indicators such as SF and sTfR.

## Figures and Tables

**Table 1:** The distribution of age and gender in three groups.

Group	Mean Age	Gender	
		Male	Female
IDA (*n* = 56)	42	9	47
NIDA (*n* = 38)	47	15	23
Control (*n* = 46)	47	16	30
Total (*n* = 140)	45	40	100
statistics	F = 2.898	χ^2^ = 27.37	
*p* value	0.059	0.025	

IDA, iron Deficiency Anemia; NIDA, non-Iron Deficiency Anemia.

**Table 2:** Comparison of hematologic and biochemical indexes for the different diagnostic groups.

Indexes	IDA Group (*n* = 56)	NIDA Group (*n* = 38)	Control Group (*n* = 46)	F	*p* Value
CHr (pg)	23.3 ± 4 *^,#^	32.5 ± 5.9 ^Δ^	31.8 ± 2.5	73.35	0.000
Hb (g/L)	82 ± 18 ^#^	87 ± 17 ^Δ^	140 ± 20	143.893	0.000
HCT (%)	28.1 ± 5.1 *^,#^	26.1 ± 5.3 ^Δ^	40.6 ± 3.8	123.27	0.000
MCV (fL)	71.6 ± 8.4 *^,#^	93.6 ± 15.4	89.7 ± 6	65.319	0.000
MCHC (g/L)	290 ± 25 *^,#^	326 ± 26 ^Δ^	339 ± 12	68.703	0.000
CH (pg)	21.2 ± 4.3 *^,#^	31.2 ± 6.4	31.1 ± 2.9	77.191	0.000
UIBC (μmol/L)	76.6 ± 32 *^,#^	30.9 ± 24.7 ^Δ^	45.7 ± 23.3	24.64	0.000
FEP (μg/gHb)	12.8 (8.5, 20.9) *^,#^	7 (4.7, 10.8) ^Δ^	3.9 (3, 5.7)	52.671	0.000
SF (ng/mL)	4.42 (3.29, 11.27) *^,#^	210.5 (114.7, 555.8) ^Δ^	102.4 (36.66, 188.3)	48.931	0.000
sTfR (mg/L)	11.02 (6.99, 14.62) *^,#^	3.26 (1.94, 6.53)	2.56 (2.01, 3.44)	57.139	0.000

CHr, reticulocyte hemoglobin content; Hb, hemoglobin; HCT, hematocrit; MCV, mean corpuscular volume; MCHC, mean corpuscular hemoglobin concentration; CH, hemoglobin content; UIBC, unsaturated iron binding capacity; FEP, free erythrocyte protoporphyrin; SF, serum ferritin; sTfR, serum transferrin receptor; MCH, mean corpuscular hemoglobin; * *p* < 0.05 between IDA group and NIDA group; ^#^ *p* < 0.05 between IDA group and Control group; ^Δ^ *p* < 0.05 between NIDA group and control group.

**Table 3:** Comparison of hematologic and biochemical indexes results for subjects with and without inflammation in IDA group and NIDA group.

Indexes	With Inflammation (*n* = 24)	Without Inflammation (*n* = 66)	*t*	*p* Value
CHr (pg)	27.1 ± 6.1	26.9 ± 6.6	0.141	0.889
Hb (g/L)	78 ± 18	86 ± 17	−1.898	0.061
HCT (%)	25.2 ± 4.8	27.9 ± 5.1	−2.233	0.028
MCV (fL)	83.8 ± 15.4	79.1 ± 15.9	1.25	0.215
MCHC (g/L)	307 ± 31	304 ± 31	0.471	0.639
CH (pg)	26.4 ± 7.1	24.9 ± 7.1	0.88	0.381
UIBC (μmol/L)	38 ± 27.4	59.9 ± 37.9	−2.133	0.037
FEP (μg/gHb)	10 (6, 17.5)	10.5 (6.8, 16.3)	−0.268	0.789
SF (ng/mL)	226.3 (8.79, 1093.75)	8.1 (3.6, 114.7)	2.813	0.008
sTfR (mg/L)	3.68 (2.15, 11.68)	7.74 (4.54, 13.02)	−1.805	0.08

CHr, reticulocyte hemoglobin content; Hb, hemoglobin; HCT, hematocrit; MCV, mean corpuscular volume; MCHC, mean corpuscular hemoglobin concentration; CH, hemoglobin content; UIBC, unsaturated iron binding capacity; FEP, free erythrocyte protoporphyrin; SF, serum ferritin; sTfR, serum transferrin receptor; MCH, mean corpuscular hemoglobin.

**Table 4:** Optimal prognostic values of different blood parameters for iron deficiency anemia according to receiver operating characteristic curve.

Indicators	Sensitivity %	Specificity %	Positive Predictive Value %	Negative Predictive Value %	Cut-Off	ROC Area
CHr (pg)	87.5	92.9	89.1	91.8	27.2	0.929
MCV (fL)	83.9	92.9	88.7	89.7	76.6	0.922
SF (ng/mL)	79.2	93.9	88.4	88.5	12.9	0.887
sTfR (mg/L)	86.8	81.7	75.4	90.5	4.89	0.900
FEP (μg/gHb)	92.6	70.0	67.6	93.3	6.4	0.873
Hb (g/L)	96.4	60.7	62.1	92.2	105	0.804

CHr, reticulocyte hemoglobin content; MCV, mean corpuscular volume; SF, serum ferritin; sTfR, serum transferrin receptor; FEP, free erythrocyte protoporphyrin; Hb, hemoglobin.
